# Patients on Losartan Have Similar Rates of Manipulation Under Anesthesia After Total Knee Arthroplasty

**DOI:** 10.7759/cureus.89572

**Published:** 2025-08-07

**Authors:** Benjamin Miltenberg, Alexander Linton, Elizabeth Abe, Brandon J Martinazzi, Gabriel Furey, William L Johns, Matthew B Sherman, James J Purtill, Eric B Smith

**Affiliations:** 1 Orthopaedic Surgery, The Rothman Orthopaedic Institute at Thomas Jefferson University, Philadelphia, USA; 2 Orthopaedics, The Rothman Orthopaedic Institute at Thomas Jefferson University, Philadelphia, USA; 3 Orthopaedics/Orthopaedic Surgery, The Rothman Orthopaedic Institute at Thomas Jefferson University, Philadelphia, USA

**Keywords:** arthrofibrosis, losartan, mua, stiffness, tka

## Abstract

Introduction

Patients have identified knee stiffness as a factor contributing to postoperative dissatisfaction after total knee arthroplasty (TKA). Losartan is an angiotensin receptor blocker (ARB) that has demonstrated antifibrotic effects; however, the impact of perioperative losartan on arthrofibrosis after TKA is not well understood. Therefore, the purpose of this study was to determine if losartan exhibits antifibrotic benefits in patients who undergo TKA by decreasing the rates of manipulation under anesthesia (MUA), when compared to patients who are not taking losartan.

Methods

All patients who underwent primary TKA by fellowship-trained arthroplasty surgeons at a single institution from January 1^st^, 2020 through December 31^st^, 2023 were identified by Current Procedural Terminology (CPT) code (27447). Patient demographic and surgery-specific information was collected. Patients were grouped into cohorts based on the presence of a patient-reported active prescription for losartan in the perioperative period. A 3:1 propensity match was performed. The two cohorts underwent matching based on angiotensin-converting enzyme inhibitor prescription, cyclooxygenase-2 (COX-2) inhibitor usage, age, sex, body mass index, and race. Postoperative rates of MUA (CPT 27570) and MUA with lysis of adhesions (LOA) (CPT 29884) were calculated, and Knee Injury and Osteoarthritis Outcome Score (KOOS), Pain Catastrophizing Scale (PCS), and Mental Component Summary (MCS) scores were collected.

Results

Twenty-seven thousand two hundred and twenty patients who underwent primary TKA within the study period were identified. Prior to propensity matching, 25,219/27,220 (92.6%) did not have a prescription for losartan within the perioperative period and 2,001/27,220 (7.4%) had a prescription for losartan within the perioperative period. After propensity matching, cohorts consisted of 6,024 patients who did not have a prescription for losartan within the perioperative period and 2,008 patients who had a prescription for losartan. There was no significant difference in the rate of MUA between patients with (74/2,008 - 3.69%) or without (240/6,024 - 3.98%) a prescription for losartan (X^2^ (1, N = 8032) = 0.3, p = 0.60). Additionally, there was no difference in the rate of MUA with LOA between patients with (12/2,008 - 0.60%) or without (18/6,024 - 0.30%) a prescription for losartan (X^2^ (1, N = 8032) = 2.9, p = 0.09). The odds ratio for MUA and MUA with LOA between groups was 1.08 (95% confidence interval 0.8 to 1.4; p = 0.55) and 0.50 (95% confidence interval 0.2-1.0p = 0.06), respectively. Additionally, there was no significant difference between groups with regard to postoperative KOOS, PCS, or MCS scores.

Conclusions

There are similar rates of MUA after primary TKA during the first postoperative year in patients with and without a prescription for losartan. However, this should be interpreted with caution as we are underpowered to detect small differences for this relatively rare outcome. Additionally, we found no difference in postoperative KOOS, PCS, and MCS between patients taking losartan and those not taking losartan. Despite the antifibrotic properties of losartan, its clinical impact on arthrofibrosis after TKA may be limited.

## Introduction

Total knee arthroplasty (TKA) has been shown to be a highly effective treatment for end-stage knee arthritis with the majority of patients who undergo TKA appreciating overall satisfaction postoperatively [1]. Consequently, the number of TKA performed in the United States will continue to increase and likely exceed 1.2 million annually by 2025 [2]. However, as many as 20% of patients who undergo TKA remain dissatisfied postoperatively [1]. Patients have identified knee stiffness, difficulty performing activities of daily living (ADLs), and lower functional performance as factors contributing to their postoperative dissatisfaction [1]. In relation to stiffness, arthrofibrosis after primary TKA occurs in approximately 4% of patients, characterized histologically by the proliferation of scar tissue and clinically by reduced range of motion (ROM), often necessitating manipulation under anesthesia (MUA) within the postoperative period [3]. Though poorly understood, arthrofibrosis is hypothesized to result from an impaired healing process in which a pro-inflammatory insult, such as TKA, stimulates the proliferation of myofibroblasts and subsequent extracellular matrix (ECM) and type-1 collagen deposition [4]. While these antifibrotic medications have been well studied in animal models, less is known regarding their utility when translated to clinical practice. To date, there are few studies that investigate the impact of ARBs on arthrofibrosis after TKA.

To mitigate the risk of postoperative stiffness following TKA, multiple studies have investigated drugs with potential antifibrotic benefits [5-9]. Specifically, angiotensin receptor blockers (ARBs) are commonly prescribed anti-hypertensive medications that have demonstrated antifibrotic effects by attenuating transforming growth factor beta (TGF-β) signaling [10,11]. While these antifibrotic medications have been well studied in animal models, less is known regarding their utility when translated to clinical practice.

To date, there are few studies that investigate the impact of ARBs on arthrofibrosis after TKA. A retrospective study conducted by Albright et al. found immediate postoperative use of ARBs to be associated with decreased rates of MUA and arthroscopic lysis of adhesions (LOA) [9]. Similarly, Rana et al. determined that patients taking ARBs, specifically Losartan, prior to TKA had significantly lower incidences of readmissions and rates of MUA [8]. While these two studies demonstrated a potentially therapeutic benefit of ARBs for the prevention of stiffness following TKA, both studies utilized large databases, which are inherently limited with regard to granularity of analysis and may impart biases secondary to missing data, incarceration data, or the statistical impart of large sample sizes [12].

Arthrofibrosis remains a challenging complication of TKA that has yet to be completely understood. We hypothesized that patients prescribed losartan for at least 90 days prior to and following TKA will have decreased rates of MUA and MUA with LOA when compared to patients not taking any form of ARB. Therefore, the purpose of this retrospective, single-institution cohort study was to determine if losartan exhibits antifibrotic benefits in patients who undergo TKA by decreasing the rates of MUA, when compared to patients who are not taking losartan.

## Materials and methods

Following Institutional Review Board review, a retrospective, single-institution query was performed. To maximize generalizability and statistical power, all patients who underwent primary TKA by fellowship-trained arthroplasty surgeons at a single institution from January 1st, 2020 through December 31st, 2023 were identified by Current Procedural Terminology (CPT) code (27447). Patients were included if they underwent primary TKA without concomitant procedures and had a minimum of one year of follow-up. Patients were excluded if they had a documented history of arthrofibrosis, if they underwent surgery with a hinge prosthesis, if they experienced an extensor mechanism disruption, or postoperative infection. Patient demographic and surgery-specific information was collected by chart review. As part of our institution's routine office visit protocol, home medication review was completed for each patient at each visit. Active medications were subsequently recorded. Subsequent MUA (27570) and MUA with LOA (CPT 29884) were searched by CPT code. Knee Injury and Osteoarthritis Outcome Score (KOOS), Pain Catastrophizing Scale (PCS), and Mental Component Summary (MCS) scores were collected in person at each visit. Preoperative KOOS scores were taken from the office visit closest to surgery, and postoperative KOOS, PCS, and MCS scores were taken from the final follow-up visit. Data on length of stay and postoperative destination were collected from hospital/facility records. 

Patients were grouped into cohorts based on the presence or absence of a preoperative losartan prescription. The losartan group included patients who had an active prescription for losartan for at least 90 days prior to and 90 days following TKA, regardless of dosage. The non-losartan group (NLG) consisted of patients without a coded prescription for losartan during the same time period. Patients were propensity matched in a three-to-one fashion using logistic regression, using nearest-neighbor matching with a tolerance level of 0.01. Characteristics with a standardized mean difference between cohorts <0.1 were considered well matched. The two cohorts underwent propensity score matching based on age, sex, body mass index, and race. Additionally, patients were matched based on angiotensin-converting enzyme inhibitor (ACEi) prescription and COX-2 inhibitor usage, as these medications have also been reported to have antifibrotic properties, which could lead to confounding.

All patients meeting inclusion/exclusion criteria within the study window were included to maximize statistical power. An a priori sample size calculator was conducted utilizing the rate of MUA after TKA in patients taking losartan (2.5%) and patients not taking losartan (2.8%) reported by Rana et al. [8]. To maintain 80% power with a 3:1 enrollment, 90,963 patients would be required in the non-losartan group, and 30,321 patients would be required in the losartan group. T-tests or Mann-Whitney U tests were used to compare continuous data, and chi-square tests were used to compare categorical data. Our alpha was set at 0.05.

## Results

**Figure 1:**
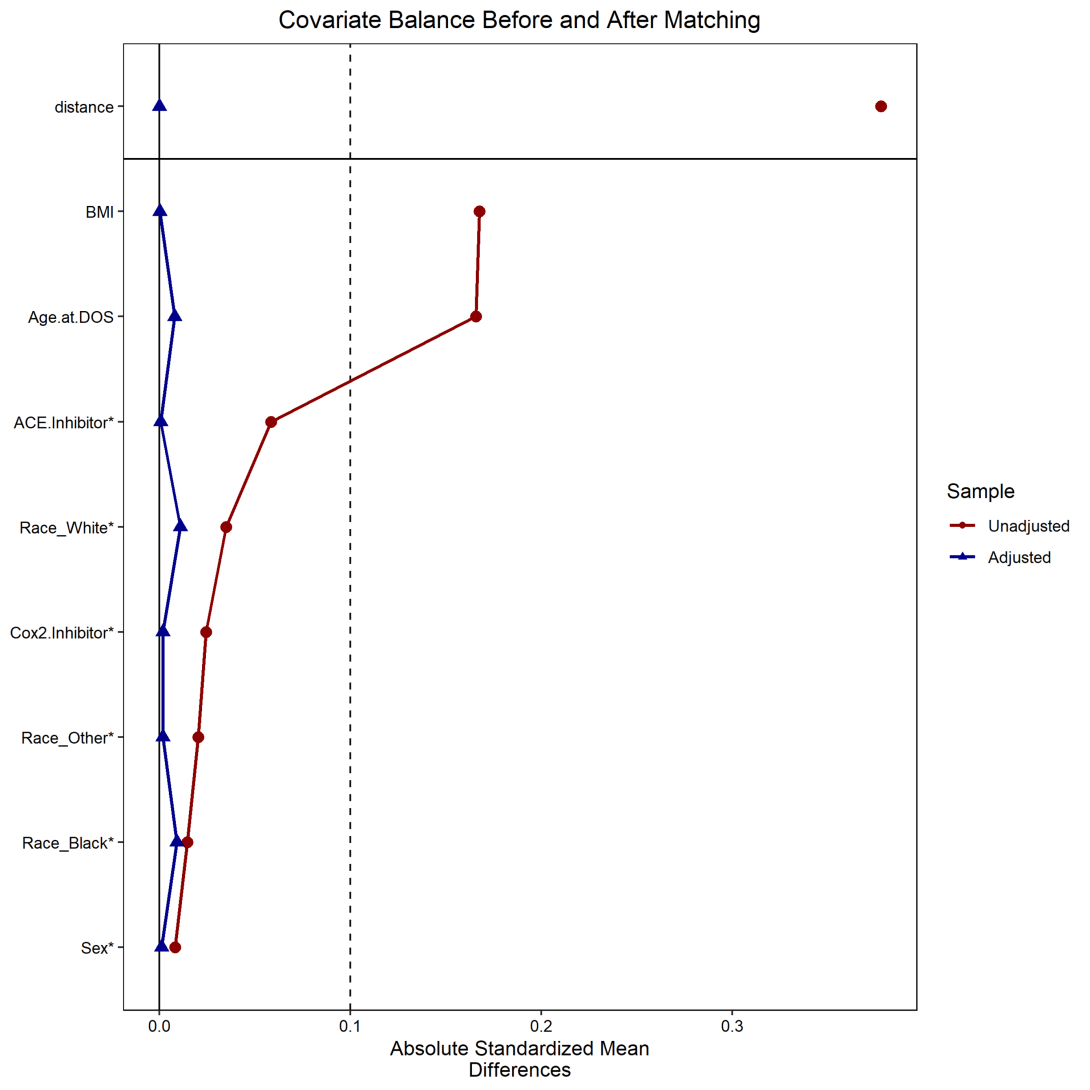
Love Plot of Covariates Before and After Matching BMI: Body mass index; ACE inhibitor: angiotensin converting enzyme inhibitor; COX-2 inhibitor: cyclooxygenase-2 inhibitor; DOS: day of surgery

Twenty-seven thousand two hundred and twenty patients who underwent primary TKA within the study period were identified. Prior to propensity matching, 25,219/27,220 (92.6%) did not have a prescription for losartan within the perioperative period and 2,001/27,220 (7.4%) had a prescription for losartan within the perioperative period. Information on patient demographics and medication use prior to matching can be seen in Table 1. After propensity matching, cohorts consisted of 6,024 patients who did not have a prescription for losartan within the perioperative period and 2,008 patients who had a prescription for losartan within the perioperative period. Information on patient demographics and medication use after propensity matching can be seen in Table 2 and Figure 1.

**Table 1:** Demographic Data Prior to Matching ACE: Angiotensin converting enzyme, {}: 95% Confidence Interval, ACE inhibitors: angiotensin-converting enzyme inhibitors, COX-2 inhibitors: cyclooxygenase-2 inhibitors T-tests or Mann-Whitney U tests were used to compare continuous data and chi-square tests were used to compare categorical data. Bold denotes statistical significance

Variables	No Losartan	Losartan	p-value
	N=25273	N=2008	
ACE Inhibitor			<0.001
No	22838 (90.4%)	1932 (96.2%)	
Yes	2435 (9.63%)	76 (3.78%)	
Cox2 Inhibitor			0.008
No	20490 (81.1%)	1677 (83.5%)	
Yes	4783 (18.9%)	331 (16.5%)	
Age	68.0 {61.0;74.0}	69.0 {63.0;74.0}	<0.001
Sex			0.476
Female	14828 (58.7%)	1195 (59.5%)	
Male	10445 (41.3%)	813 (40.5%)	
Body Mass Index	30.8 {27.2;35.0}	31.8 {28.1;35.9}	<0.001
Race			0.002
White	18607 (73.6%)	1408 (70.1%)	
Black	2132 (8.44%)	199 (9.91%)	
Other	4534 (17.9%)	401 (20.0%)	

**Table 2:** Matching Results ACE inhibitor: Angiotensin converting enzyme inhibitor; COX-2 inhibitors: cyclooxygenase-2 inhibitors; DOS: day of surgery; {}: 95% confidence interval Patients were propensity matched in a three-to-one fashion using logistic regression, using nearest-neighbor matching with a tolerance level of 0.01. Characteristics with a standardized mean difference between cohorts <0.1 were considered well matched.

Variables	No Losartan	Losartan	p value	Standard Mean Difference
	N=6024	N=2008		
ACE Inhibitor			0.919	0.0043
No	5801 (96.3%)	1932 (96.2%)		
Yes	223 (3.70%)	76 (3.78%)		
Cox2 Inhibitor			0.875	0.0049
No	5042 (83.7%)	1677 (83.5%)		
Yes	982 (16.3%)	331 (16.5%)		
Age at DOS	69.0 {68.8-69.2}	69.0 {63.0;74.0}	0.882	-0.0081
Sex			0.948	
Female	3592 (59.6%)	1195 (59.5%)		0.0024
Male	2432 (40.4%)	813 (40.5%)		-0.0024
Body Mass Index	31.8 {28.1;35.9}	31.8 {28.1;35.9}	0.917	0.0004
Race			0.427	
White	4291 (71.2%)	1408 (70.1%)		0.0311
Black	541 (8.98%)	199 (9.91%)		0.0046
Other	1192 (19.8%)	401 (20.0%)		-0.0243

Additional data on patient demographic and medical comorbidities after matching can be seen in Table 3. There was a significant difference in the location of procedure with patients taking losartan more likely than patients not taking losartan to undergo surgery at an inpatient facility. Additionally, patients taking losartan were more likely to have diabetes, hypertension, and renal disease which are medical comorbidities for which losartan may be prescribed.

**Table 3:** Additional Patient Information ASA: American Society of Anesthesiologists Score; {}: standard deviation T-tests or Mann-Whitney U tests were used to compare continuous data and chi-square tests were used to compare categorical data. Bold denotes statistical significance

Variables	No Losartan	Losartan	p-value
Laterality			0.545
Bilateral	1 (0.02%)	1 (0.05%)	
Left	2932 (48.7%)	985 (49.1%)	
Right	3090 (51.3%)	1022 (50.9%)	
Inpatient vs. Outpatient			<0.001
Inpatient	2282 (37.9%)	1001 (49.9%)	
Outpatient	3741 (62.1%)	1007 (50.1%)	
ASA			0.063
1	9 (0.48%)	3 (0.42%)	
2	921 (49.0%)	308 (43.4%)	
3	931 (49.5%)	387 (54.6%)	
4	20 (1.06%)	11 (1.55%)	
Elixhauser Comorbidity Index	1.07 {1.32}	1.20 {1.33}	0.015
Heart Failure			0.696
No	1845 (97.3%)	695 (96.9%)	
Yes	51 (2.69%)	22 (3.07%)	
Peripheral Vascular Disease			0.243
No	1868 (98.5%)	701 (97.8%)	
Yes	28 (1.48%)	16 (2.23%)	
Hypertension			<0.001
No	1339 (45.8%)	366 (30.2%)	
Yes	1584 (54.2%)	847 (69.8%)	
Chronic Obstructive Pulmonary Disease			0.261
No	1682 (88.7%)	624 (87.0%)	
Yes	214 (11.3%)	93 (13.0%)	
Diabetes			0.012
No	1697 (89.5%)	616 (85.9%)	
Yes	199 (10.5%)	101 (14.1%)	
Hypothyroidism			0.852
No	1667 (87.9%)	633 (88.3%)	
Yes	229 (12.1%)	84 (11.7%)	
Renal Failure			0.002
No	1764 (93.0%)	640 (89.3%)	
Yes	132 (6.96%)	77 (10.7%)	
Coagulopathy			0.703
No	1878 (99.1%)	712 (99.3%)	
Yes	18 (0.95%)	5 (0.70%)	
Anesthesia			0.085
General	15 (1.98%)	12 (4.10%)	
Spinal	742 (98.0%)	281 (95.9%)	

MUA was required in 240/6024 (3.98%) of patients without a prescription for losartan and 74/2008 (3.69%) of patients with a prescription for losartan (X2 (1, N = 8032) = 0.3, p = 0.60). MUA with LOA was required in 18/6024 (0.30%) of patients without a prescription for losartan and 12/2008 (0.60%) of patients with a prescription for losartan (X2 (1, N = 8032) = 2.9, 0.09). The odds ratio for MUA and MUA with LOA between groups was 1.08 (95% confidence interval 0.8 to 1.4; p = 0.55) and 0.50 (95% confidence interval 0.2-1.0p = 0.06), respectively. Patients taking losartan had longer lengths of stay compared to patients not taking losartan (1.34 days vs 1.28 days; p < 0.001). There was no statistically significant difference in discharge destination. Additionally, there was no difference in postoperative KOOS, PCS, or MCS. Outcomes data can be seen in Table 4.

**Table 4:** Outcomes {}: 95% Confidence Interval; KOOS: Knee Injury and Osteoarthritis Outcome Score; PCS: Pain Catastrophizing Scale; MCS: Mental Component Summary Score; Pre-Op: preoperative; Post-Op: postoperative; MUA: manipulation under anesthesia; LOA: lysis of adhesions T-tests or Mann-Whitney U tests were used to compare continuous data and chi-square tests were used to compare categorical data. Bold denotes statistical significance

Variables	No Losartan	Losartan	p value
	N = 6024	N = 2008	
Length of Stay (Days)	1.28 {1.2-1.3}	1.34 {1.3-1.4}	<0.001
Discharge Destination			0.141
Another Hospital	1 (0.02%)	0 (0.00%)	
Expired	2 (0.04%)	0 (0.00%)	
Home	1968 (35.4%)	690 (38.6%)	
Home Healthcare	3329 (59.8%)	1009 (56.5%)	
Inpatient Rehab	52 (0.93%)	14 (0.78%)	
Skilled Nursing Facility	214 (3.84%)	74 (4.14%)	
Pre-Op KOOS	46.6 {46.2-47.0}	47.0 {46.4-47.6}	0.545
Post-Op KOOS	71.4 {71.0-71.8}	70.7 {70.0-71.4}	0.381
Pre Op PCS	33.8 {33.6-34.0}	33.7 {83.3-34.1}	0.918
Post Op PCS	41.6 {41.3-41.9}	41.4 {41.0-41.8}	0.812
Pre Op MCS	50.2 {50.0-50.5}	51.2 {50.7-51.7}	0.175
Post Op MCS	53.8 {53.6-54.0}	54.6 {54.2-55.0}	0.721
MUA			0.595
No	5784 (96.0%)	1934 (96.3%)	
Yes	240 (3.98%)	74 (3.69%)	
MUA with LOA			0.091
No	6004 (99.7%)	1996 (99.4%)	
Yes	18 (0.30%)	12 (0.60%)	

## Discussion

The principal finding of the study is that there was no significant difference in the rate of MUA after primary TKA in patients taking losartan and those not taking losartan. Additionally, we found no difference in postoperative KOOS, PCS, and MCS between patients taking losartan and those not taking losartan. While postoperative stiffness remains a challenge after TKA, this study suggests that the antifibrotic effects of losartan may not result in a clinically meaningful impact on postoperative joint stiffness.

Stiffness or arthrofibrosis is an infrequent yet challenging issue following TKA. The rate of postoperative arthrofibrosis after TKA is reported to be between four-16% and treatment predominantly revolves around mechanical interventions like aggressive physical therapy and MUA [3,13,14]. While there is no commonly accepted pharmacologic intervention for either prophylaxis or treatment, there are many molecular targets in the well-described pro-fibrotic pathway [15-19]. Perhaps the most well-studied target in the orthopaedic literature is angiotensin II [11,20,21]. Angiotensin II is a critical component of the profibrotic cascade, activating numerous JAK/STAT pathways as well as many profibrotic proteins [15-18,22]. For this reason, drugs like losartan, which function to inhibit angiotensin II, have received attention as potential mediators of postoperative stiffness [15-18,22]. At the molecular level, multiple studies have shown losartan’s impact on the fibrotic quality of tissue and the molecular expression of profibrotic molecules [6,22-25]. Macroscopically, studies have shown losartan to improve the range of motion and decrease the formation of adhesions in large joints in animal models [6,22-25].

The clinically based data on the impact losartan has on postoperative stiffness after TKA are conflicting. Utilizing the TriNetX database, Rana et al. found that TKA patients with a perioperative prescription for losartan had lower odds of undergoing MUA within one year of their index surgery compared to patients without a perioperative prescription for losartan (OR 0.87; p = 0.009) [8]. There was a rate of MUA of 2.5% for patients with a perioperative prescription for losartan and a rate of 2.8% for patients without a perioperative prescription for losartan [8]. This would equate to a number needed to treat of 334. Similarly, utilizing the PearlDiver database, Albright et al. found that patients who filled perioperative prescriptions for losartan following TKA how a lower adjusted odds ratio of MUA when compared with patients without a prescription for ARB (adjusted OR 0.94; p = 0.016) [9]. The rate of MUA for patients with a prescription for an ARB was 2.76% while the rate for the control group was 3.84% with a number needed to treat of 93 [9]. Additionally, using an employer-sponsored healthcare database (Truven Marketscan), Premkumar et al. found that perioperative use of losartan was associated with a decreased odds of MUA (OR 0.80; p = 0.007) [6]. While these large, database studies have shown a statistically significant difference in MUA, other studies have called into question the clinical impact losartan may have in the TKA patient. For example, Arraut et al. found that the postoperative range of motion and rate of MUA were similar for patients taking losartan and those not taking losartan [24]. Likewise, Hernandez et al. found no association between angiotensin receptor blocker prescription and postoperative rates of MUA or arthrofibrosis in TKA patients [26]. Overall, these studies offer a conflicting picture of the clinical impact losartan may have on TKA patients.

Our findings, which sample over 8,000 patients from a single institution, highlight that any difference in the rate of MUA after TKA imparted by losartan may not have a clinically or statistically significant impact. While prior large database studies have described a statistically significant difference in the rate of MUA after TKA, this may be imparted due to the statistical bias associated with massive sample size. Indeed, the studies showing a statistically significant impact of losartan on MUA after TKA all came from databases with study populations ranging from roughly 50,000-1,300,000. These large sample sizes allow for very small differences between groups to appear significant. Therefore, the clinical significance of these findings must be scrutinized. This, however, has to be balanced against the need for appropriate statistical power, which is a limitation of our study, as is the case for prior single-center studies on this topic [24]. Our a priori power analysis suggested that to detect a 0.3% difference in the rate of MUA after TKA and maintain 80% power with a 3:1 enrollment, 90,963 patients would be required in the non-losartan group, and 30,321 patients would be required in the losartan group. While powering this study is likely beyond the capacity of any single institution, it further highlights the small, if present, difference imparted by perioperative losartan on stiffness after TKA. 

To help control for confounding variables, we chose propensity matching for our cohorts. However, there are innumerable medications with purported antibiotic properties, including chemotherapeutic agents, antibiotics, cardiac medications, and immunosuppressants [5,27,28]. While we propensity matched for the most commonly discussed/researched antifibrotics in the orthopaedic literature, there are other common medications that we did not match for. These include statins, antihistamines, and oral steroids [5,27,28]. Given the massive number of medications with antifibrotic properties, we were unable to control for all of these variables. This, however, introduces the risk of confounding in our study, given the fact that even after propensity matching, our cohorts were not identical with regard to medical comorbidities. Indeed, after matching, there is a significant difference in the Elixhauser Comorbidity Index, hypertension, diabetes, and renal failure between groups. While this is unsurprising given that losartan is frequently prescribed for hypertension or diabetic nephropathy, it is possible (if not likely) that these patients would have higher rates of other antibiotic medication prescriptions, particularly statins. While this does introduce additional cofounding variables to our study, these would, if anything, make the losartan cohort less prone to postoperative arthrofibrosis.

Our study must be interpreted within the context of limitations. First and foremost, there are numerous variables other than a losartan prescription that could impact the rate of postoperative MUA. While we attempted to control for cofounding variables with propensity matching, it would be impossible to control for every variable. Indeed, our post-match demographic data does suggest that patients with a losartan prescription were, on average, less healthy than control patients. While this is not suppressing, this does raise the risk of cofounding as discussed above. Moreover, our study includes all patients who underwent TKA in our practice regardless of practice pattern (cruciate retaining vs. posterior stabilized, implant technique-tibial slope, etc.) or patient characteristics (location, access to care, etc.), which again introduces more cofounding variables; however, we anticipate that these would likely be evenly split between groups. Moreover, losartan dosage, administration schedule, and titration protocols were not standardized across groups. Perhaps the only way one could truly create a homogeneous group would be through a single-surgeon study; however, this would make powering the research nearly impossible. Furthermore, modifying losartan dosage is outside the scope of orthopaedic practice and is likely not clinically or ethically justifiable for research at this time. The existing literature on this topic consists of large database studies (which are perhaps more prone to confounding) and small single-center studies. Therefore, while we are not able to control for every variable, our study provides increased statistical power while maintaining some ability to control for heterogeneity. Next, postoperative range of motion is inconsistently recorded, and therefore, was not able to be included in the study. It is possible that losartan does have an impact on range of motion, but not enough impact to affect rates of MUA. Additionally, postoperative physical therapy is part of many surgeons’ standard postoperative protocol, while other surgeons prescribe physical therapy only to patients meeting certain criteria regarding mobility/range of motion. Therefore, we were unable to assess whether losartan impacts the duration/rate of prescription of physical therapy. Additionally, we are underpowered to detect small differences in rates of MUA. This is highlighted by our power analysis which suggests we would need an order of magnitude greater enrollment in order to detect a 0.3% difference in the rate of MUA. However, we are the largest single-center study on this topic. Given the large volume of patients required to be appropriately powered for such a small difference in outcomes, a multicenter trial may be required. We did not account for the dose of losartan, which may play a role in postoperative stiffness. Bedair et al. did find that there was a dose-dependent relationship between ARBs and fibrotic tissue formation. It is possible that the standard prescription of losartan for hypertension may not maximize the anti-fibrotic effects of this medicine [11]. However, utilizing the standard prescription of losartan for medical comorbidities helps provide some generalizability to our findings. Additionally, our study was performed as a retrospective analysis, introducing selection bias to our findings. We attempted to control for this by matching for comorbidities.

## Conclusions

There are similar rates of MUA after primary TKA during the first postoperative year in patients with and without a prescription for losartan. However, this should be interpreted with caution as the we are underpowered to detect small differences for this relatively rare outcome. Additionally, we found no difference in post operative KOOS, PCS, and MCS between patients taking losartan and those not taking losartan. Despite the antifibrotic properties of losartan, its clinical impact on arthrofibrosis after TKA may be limited.
